# *Acetylcholinesterase* Inhibition by Biofumigant (Coumaran) from Leaves of *Lantana camara* in Stored Grain and Household Insect Pests

**DOI:** 10.1155/2014/187019

**Published:** 2014-06-15

**Authors:** Yallappa Rajashekar, Anjanappa Raghavendra, Nandagopal Bakthavatsalam

**Affiliations:** ^1^Animal Bioresources Programme, Institute of Bioresources and Sustainable Development, Department of Biotechnology, Government of India, Takyelpat, Imphal, Manipur 795001, India; ^2^Division of Insect Ecology, National Bureau of Agriculturally Important Insects, Bangalore, Karnataka 560024, India

## Abstract

Recent studies proved that the biofumigants could be an alternative to chemical fumigants against stored grain insect pests. For this reason, it is necessary to understand the mode of action of biofumigants. In the present study the prospectus of utilising *Lantana camara* as a potent fumigant insecticide is being discussed. Inhibition of acetylcholinesterase (AChE) by Coumaran, an active ingredient extracted from the plant *L. camara*, was studied. The biofumigant was used as an enzyme inhibitor and acetylthiocholine iodide as a substrate along with Ellman's reagent to carry out the reactions. The *in vivo* inhibition was observed in both dose dependent and time dependent in case of housefly, and the nervous tissue (ganglion) and the whole insect homogenate of stored grain insect exposed to Coumaran. The possible mode of action of Coumaran as an *acetylcholinesterase* inhibitor is discussed.

## 1. Introduction

Acetylcholine (Ach) is one of the major molecules by which nerve impulses are transmitted from nerve cell or involuntary muscle [1]. Acetylcholinesterase AChE is an enzyme that breaks down the neurotransmitter acetylcholine at the synaptic cleft (the space between two nerve cells) so the next nerve impulse can be transmitted across the synaptic gap [2]. The phosphine, organophosphates, and carbamates act by interfering with the passage of impulses in the insect nervous system [3]. Organophosphate insecticides are generally regarded as irreversible inhibitors of the enzyme acetylcholinesterase. The inability of phosphorylated AChE to hydrolyse acetylcholine, the build up of concentration of the acetylcholine in the synapse and excessive neuro excitation are the results of prolonged binding of ACh to its postsynaptic receptor. The signs of intoxication include restlessness, hyperexcitability, tremors, convulsions and paralysis leading to death [4, 5].

Stored grain insects cause extensive damage in stored wheat, rice, pulses and other commodities [6, 7]. Synthetic insecticides especially fumigants such as methyl bromide and aluminium phosphide are commonly used to manage these pests. Even though this method is effective, repeated use of these chemicals may cause environmental hazards and various biochemical changes in nontarget animals [8]. It was also reported that the insects have developed resistance against these fumigants [9]. Due to the possible role of methyl bromide in depletion of ozone layer, its use as a fumigant is banned [10]. Thus, there is a need to develop cheaper and safer alternative measures including plant based products against stored grain and household insect pests [11–13].

In this perspective, properties of plant products including essential oils and their bioactive molecules have been broadly studied for the control of stored grain pests [1, 14–16]. The properties include toxic, ovicidal, repellent, antifeedant and other properties [17]. Essential oils, allelochemicals and their individual constituents have been known to play an important role as protectants of stored grains and proved to possess repellent and insecticidal properties [18]. The different organic extracts from root powder of* Decalepis hamiltonii* (Wight and Arn) and the bioactive compounds from Decalesides showed potential to be used as grain protectants against grain insect pests [14, 19]. Many plant secondary metabolites such as monoterpenoids, polyphenols, and sugars have insecticidal activity against stored grain insect pests [7, 20]. However, the detailed studies on the biochemical effects of these compounds on insect physiology would reveal the affected target sites in the respective pests.

Several reports indicate that monoterpenoids and most of the plant volatiles cause insect mortality by inhibiting acetylcholinesterase enzyme [1]. The monoterpenoid (1,8-cineole) was reported to be potent AChE inhibitor [21]. The action of essential oils and biofumigants on insects could be neurotoxic based on behavioural symptoms similar to those produced by organophosphates [22] or inhibition of AChE [23]. Evidently, the studies on mode of action of biofumigants in insects have largely focused on monoterpenoids. So, it becomes very important to study the mode of other compounds showing fumigant action.

*Lantana camara *(Verbenaceae), an erect shrub, grows widely throughout the tropical, subtropical and temperate parts of the world. Earlier work showed that the leaves of* L. camara *were the source of insecticidal activity [24]. Previously, we investigated the insecticidal activity of extracts from leaves of* L. camara *against the storage pests, namely, rice weevil,* Sitophilus oryzae *L. (Coleoptera: Curculionidae) rust red flour beetle,* Tribolium castaneum *Herbst. (Coleoptera: Tenebrionidae), and adzuki bean weevil,* Callosobruchus chinensis *Fab. (Coleoptera: Bruchidae) [25]. The present study aims to investigate the mode of action of Coumaran in the inhibition of acetylcholinesterase.

## 2. Materials and Methods

### 2.1. Insects

Cultures of* S. oryzae* were maintained on whole wheat (*Triticium aestivum*), whereas* T. castaneum* were reared on whole wheat flour with 2% yeast powder. Houseflies (*Musca domestica)* were reared in a mixture of sterilized bran, milk powder and water. The adults were allowed free access to water and thick paste of condensed milk and milk powder. Cultures were maintained at 30 ± 1°C and 70% relative humidity. Adults of* S. oryzae* (3–5 d) and other species (2-3 d) were used for the experiments. Experiments were carried out in the laboratory at 27 ± 2°C and 70 ± 5% r.h. [14, 25].

### 2.2. Chemicals

AChE, acetylthiocholine iodide, Coumaran, and Pyridostigmine bromide were procured from Sigma chemical Co. (St. Louis, MO, USA). Ammonium molybdate, ascorbic acid, trichloroacetic acid (TCA), hydrochloric acid (HCl) and other chemicals were procured from Sisco Research Laboratory, Mumbai, India.

### 2.3. Biofumigant Preparation

Healthy, mature leaves of* L. camara *were collected from Hesaraghatta, Bangalore, India. The leaves were cut into small pieces, shade-dried and powdered. One hundred gram of leaf powder was sequentially extracted with a series of solvents of increasing polarity, namely, hexane, ethyl acetate, acetone and methanol in a Soxhlet apparatus. The solvent was evaporated* in vacuo *and the residue was dissolved in a known volume of methanol. This solution was screened for insecticidal activity using contact/fumigant toxicity bioassay. Since the methanolic extract was found to show the highest insecticidal activity in the preliminary screening, this was selected for the isolation of the biofumigant [25].

### 2.4. Inhibition of AChE

*In vivo* inhibition of AChE in relation to the toxicity of Coumaran was investigated in both houseflies and stored grain pests.

*Dose-Response Study*. Insects were exposed to LC_25_, LC_50_, and LC_90_ doses of Coumaran in the fumigant bioassay [25]. The doses were selected based on the results of toxicity of Coumaran to stored grain insects and housefly. Respective batches of solvent treated controls were also employed. After 45 min exposure insects were removed. In case of houseflies the head and thorax were dissected out and frozen for enzyme assay, where as in case of stored grain insects, whole insect was homogenized and stored at −20°C for enzyme assay. 

*Time-Course Study*. Insects were exposed to single LC_50_ dose of Coumaran in the fumigation bioassay and removed at various exposure time intervals (15, 30, 45, and 60 min). Solvent (ethanol) treated groups served as control. After various time intervals of exposure, insects were removed and the insect tissues were dissected as described above for enzyme assay.

Inhibition of acetylcholinesterase (AChE) was estimated by colorimetric method previously described by Ellman et al. [26]. The reaction was carried out in a cuvette by dissolving Coumaran in absolute ethanol to which 40 *μ*L of substrate (acetylthiocholine iodide) was added and then DTNB (200 *μ*L) followed by inhibitor (Pyridostigmine bromide) solution (1 mL) in different concentrations of 0.01 *μ*M, 0.1 *μ*M, 0.5 *μ*M, and 1 *μ*M. Tests and control assays (without Coumaran) were corrected by blanks for nonenzymatic hydrolysis. Each assay was replicated only twice since the results from the second replication were practically similar to those produced by the first replication. Level of AChE activity was estimated by using Shimadzu UV-1600 Spectrophotometer at 412 nm and at 25°C.

#### 2.4.1. *In Vitro* Inhibition of Acetylcholinesterase

In* in vitro* inhibition of acetylcholinesterase by Coumaran in the head and thorax of houseflies, whole insect homogenate of stored grain insects was studied. The enzyme was preincubated with Coumaran (0.01 *μ*M–1 *μ*M) at 37°C for 30 min and the inhibition of AChE was determined. LC_50_ were calculated by regression analysis.

Protein content was measured by the method of Lowry et al. [27] using BSA as the standard.

### 2.5. Statistical Analysis

The data obtained from the studies was analysed using one-way ANOVA at (*P* < 0.01) and mean values were separated by using Tukey and Statplus 2007 software. The data was expressed as mean ± SD. Probit analysis was performed for calculating LC_25_, LC_50_, and LC_90_ [28].

## 3. Results

### 3.1. *In Vivo* Inhibition of Acetylcholinesterase in relation to Insect Toxicity

**Figure 1:**
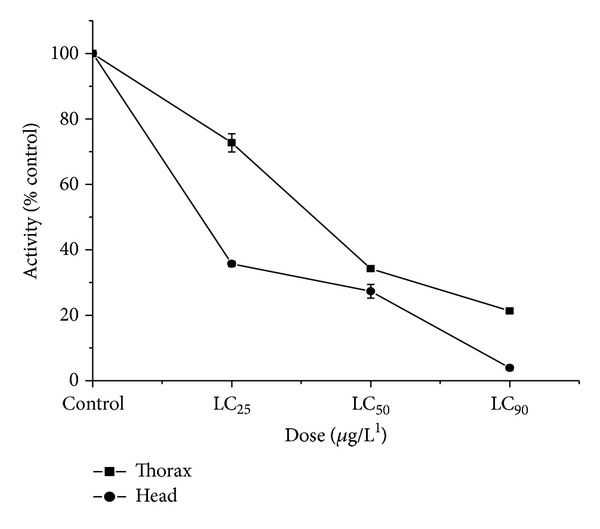
Dose-dependent inhibitions* in vivo* of AchE in the housefly by Coumaran at 45 min of exposure by fumigation (control activity: head homogenate = 178.03 micromoles of substrate hydrolyzed/minute/mg of protein; thorax homogenate = 169.14 micromoles of substrate hydrolyzed/minute/mg of protein) (*n* = 6, error bars, standard deviation).

**Figure 2:**
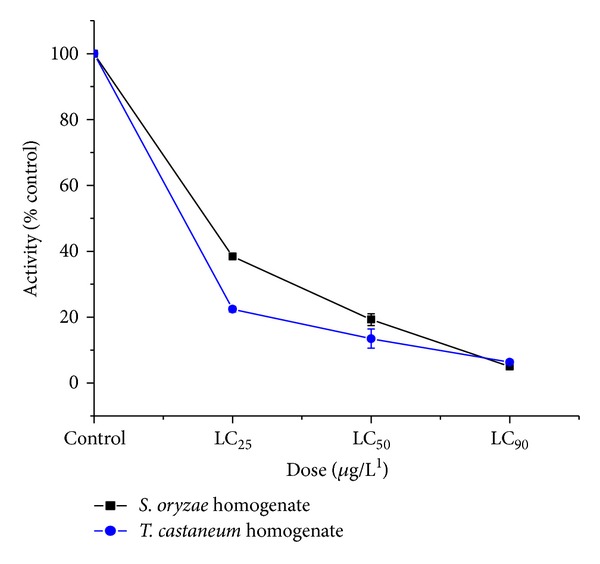
Dose-dependent inhibitions* in vivo* of AchE in the homogenate of* S. oryzae *and* T. castaneum* by Coumaran at 45 min of exposure by fumigation (control activity:* S. oryzae* homogenate = 314.6 micromoles of substrate hydrolyzed/minute/mg of protein;* T. castaneum* homogenate = 348.8 micromoles of substrate hydrolyzed/minute/mg of protein) (*n* = 6, error bars, standard deviation).

*Dose Response*. Activity of acetylcholinesterase in insects exposed to LC_25_, LC_50_, and LC_90_ doses of Coumaran was markedly inhibited in dose-dependent studies in the head and thorax of housefly (Figure 1) and homogenate of stored grain insect pests (Figure 2). The* in vivo *enzyme inhibition was dose dependent and correlated with the knockdown effect measured at 45 min of exposure in the fumigation bioassay.

**Figure 3:**
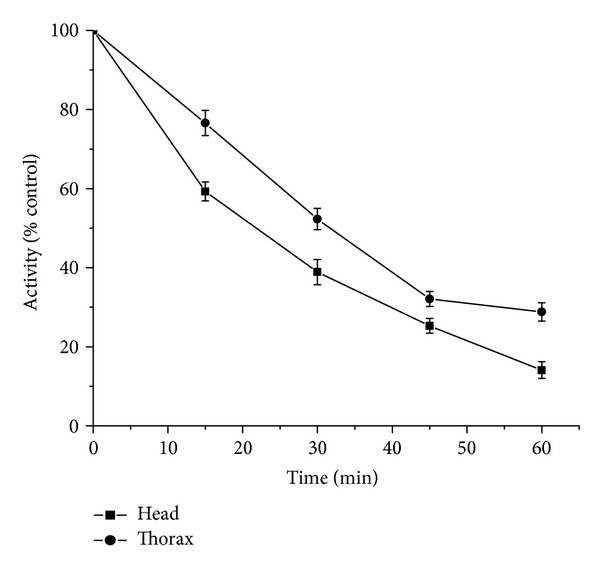
Time-course of* in vivo* inhibition of AchE in relation to knockdown of houseflies treated with Coumaran at LC_50_ concentration (0.032 *μ*g/L) by fumigation (control activity: head homogenate = 188.7 micromoles of substrate hydrolyzed/minute/mg of protein; thorax homogenate = 166.5 micromoles of substrate hydrolyzed/minute/mg of protein) (*n* = 6, error bars, standard deviation).

**Figure 4:**
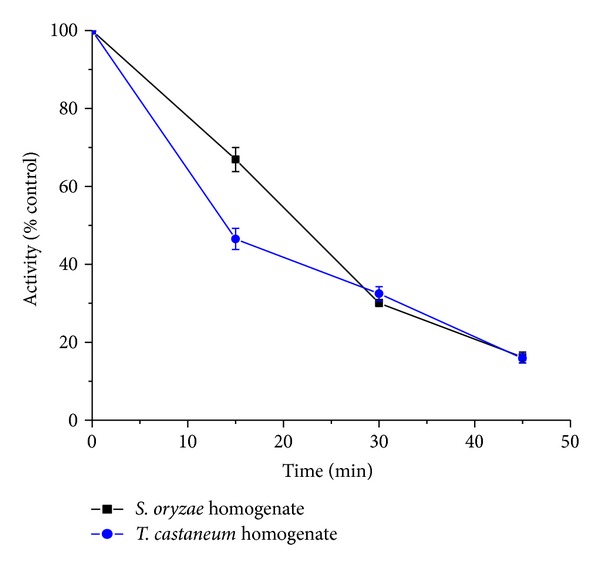
Time-course of* in vivo* inhibition of AchE in relation to knockdown of stored grain beetles treated with Coumaran at LC_50_ concentration (0.032 *μ*g/L) by fumigation (control activity:* S. oryzae* homogenate = 304.6 micromoles of substrate hydrolyzed/minute/mg of protein;* T. castaneum* homogenate = 328.2 micromoles of substrate hydrolyzed/minute/mg of protein) (*n* = 6, error bars, standard deviation).

*Time-Course Study*. In houseflies treated with a single LC_50_ dose of Coumaran at various exposure times (0–45 min), inhibition of acetylcholinesterase increased with time and correlated with the knockdown effect (Figures 3 and 4).

### 3.2. *In Vitro* Inhibition of Acetylcholinesterase

**Figure 5:**
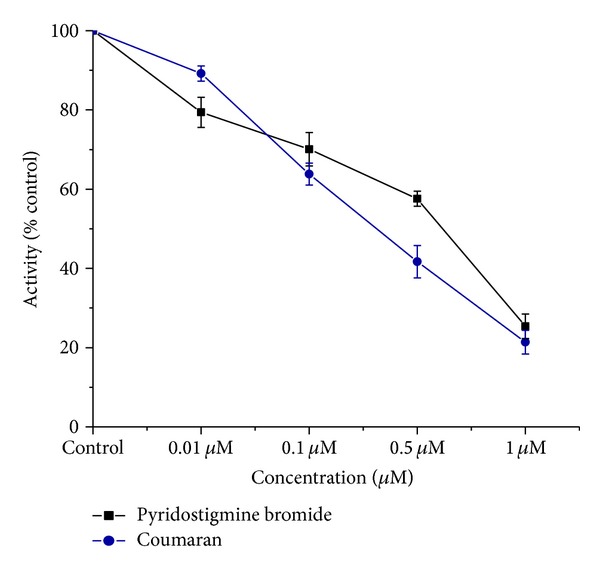
*In vitro* inhibition of AchE by Coumaran in comparison with that of Pyridostigmine bromide in houseflies (control activity: head homogenate = 238.3 micromoles of substrate hydrolyzed/minute/mg of protein) (*n* = 6, error bars, standard deviation).

**Figure 6:**
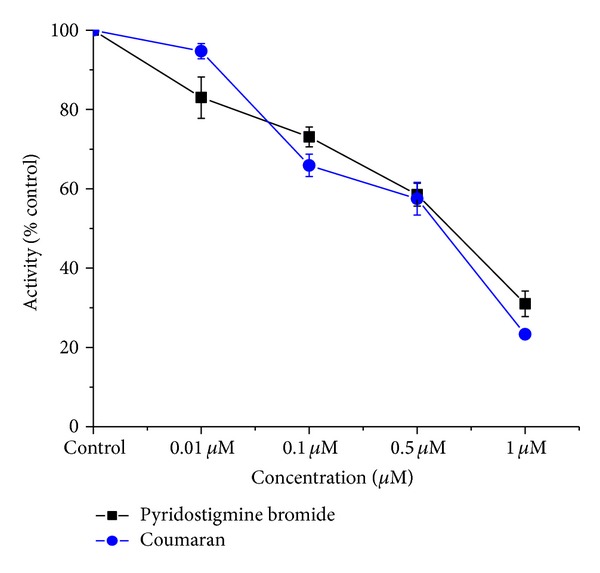
*In vitro* inhibition of AchE by Coumaran in comparison with that of Pyridostigmine bromide in houseflies (control activity: thorax homogenate = 249.1 micromoles of substrate hydrolyzed/minute/mg of protein) (*n* = 6, error bars, standard deviation).

The* in vitro* inhibition of acetylcholinesterase was increased with the concentration of Coumaran in the housefly (head and thorax) homogenate (Figures 5 and 6).

## 4. Discussion

Due to environmental concerns, health hazards to human beings and the development of resistance in insect pests to the various insecticides, constant efforts were made to discover newer insecticides both from natural sources and by chemical synthesis. The majority of insecticides have mode of action that can be classified into three categories, namely, neurotoxins, respiratory inhibitors and growth regulators [29, 30]. Most of the chemical insecticides involve target sites in the nervous system, namely, acetylcholinesterase (organophosphate and carbamate compounds), voltage-gated sodium channels (pyrethroids and organochlorines), and the acetylcholine receptor (neonicotinoids) [31–33]. Although, insect control agents (e.g., azadirachtin, JH analogues and ecdysone antagonists) acting on hormone system such as insect growth regulators have been developed, due to their lack of contact and fumigant toxicity they are not highly successful, however they do found a place in integrated pest management scheme.

Although our experimental evidence indicates that the action of Coumaran involves the respiratory pigments possibly acting on the spiracles, the insects became hyperactive indicating neural excitation which is followed by knockdown effect and mortality which remains puzzling. The symptoms excitation, respiratory spiracles and knockdown of the insects upon fumigation, point out the affected targets as neural/neuromuscular sites. However, we investigated the possible involvement of two biochemical targets critical in the transmission of nerve impulse, namely, the enzymes, acetylcholinesterase and Na^+^, K^+^ ATPase. Acetylcholinesterase (AChE), involved in the synaptic transmission of nerve impulse, is the target for insecticides belonging to the organophosphorus and carbamate group [31, 33]. Preliminary experiments showed no effect of Coumaran on Na^+^, K^+^ ATPase in insects exposed to Coumaran (data not shown).

Present study showed that AChE is inhibited severely in insects (both housefly and stored grain insects) exposed to Coumaran in the fumigation bioassay. This* in vivo* inhibition closely correlates (*r*⩾0.9) with the insect toxicity of Coumaran in dose-response and time-course studies. The* in vivo* inhibition was seen in both head and thorax in the case of housefly and the nervous tissue (ganglion) and whole insect homogenate of stored grain insect exposed to Coumaran.

Our results showed that the insects exposed to Coumaran are initially hyperactive indicating neural excitation, which is followed by knockdown effect, a symptom similar to that of insects exposed to carbamate suggesting a neurotoxic effect. The fundamental difference, however, is that the Coumaran action is mediated by fumigation with respiratory spiracles, unlike that of carbamate which acts by contact at any point of the body surface of insects. It is known that carbamates act by interfering inhibition of AChE; the carbamylated enzyme is no longer capable of affecting the hydrolysis of ACh; this results in a buildup of the neurotransmitter at a nerve synapse [4]. The toxicity of Coumaran may be attributed to its ability to inhibit the activity of AChE, which catalyses the hydrolysis of the neurotransmitter Ach at nerve synapses and neuromuscular junctions. The heterobicyclics, formate esters, Coumaran and their derivatives are the most neurologically and toxicologically active of the prototype materials [30, 34]. Another probable reason could be that the compounds present in these prototypes are neurologically active compounds that have broad impact across the nervous system which is attenuated by modified acetyl choline and acetate function. These results are in accordance with our earlier work on fumigant toxicity of Coumaran against stored grain insect pests. Therefore, we foresee potential of this biofumigant to be used in organic protection of stored commodities.

## 5. Conclusion

In the present study Coumaran was found to be a potent biofumigant isolated from leaves of* L. camara* which can be used as a biopesticide. Coumaran acts on the respiratory pigments of the insect and can be used as a biofumigant. The probable reason could be AChE inhibition at the cholinergic synapses of the insects. Further toxicity of Coumaran (if any) to the nontargeted animals is to be studied. Due to insect selectivity and from natural origin Coumaran can be used as a biofumigant against the stored grain pests.
